# On the response of the Baltic proper to changes of the total phosphorus supply

**DOI:** 10.1007/s13280-017-0933-7

**Published:** 2017-07-19

**Authors:** Anders Stigebrandt

**Affiliations:** Department of Marine Sciences, University of Gothenburg, Box 461, SE-40530 Gothenburg, Sweden

**Keywords:** Anoxic conditions, Baltic Sea, Internal source, P, Phosphorus model, Restoration

## Abstract

Using a time-dependent phosphorus (P) budget model for the Baltic proper, describing sources and sinks at the external borders of the water column, one may compute the e-folding time *T* of the adjustment of the winter surface water P concentration *c*_1_ to abruptly changed total P supply. The restoration time TR = 3*T* is introduced as a practical measure of the time it takes to achieve 95% of the change of *c*_1_ towards the final, equilibrium, state *c*_1e_. The P budget model, including an internal source emanating from deep anoxic bottoms, also shows that *c*_1e_ is proportional to the total P supply to the water column. About 70% of present time total P supply to the Baltic proper comes from deep anoxic bottoms. If deep bottoms were kept oxygenated, this internal P supply would be turned off and the equilibrium concentration c_1e_ would be reduced by about 70%. This should imply that the Baltic proper may be restored to a state determined by the external P supplies from land-based and oceanic sources. According to the model, restoration would take 10–15 years. Thereafter most of the equipment used for oxygenation may be shut off since also the deepwater oxygen demand by decomposition of fresh organic matter, would have decreased by about 70% implying that the deepwater would be kept oxic by the natural vertical circulation. The model presented in this paper provides a new science-based solution of the eutrophication problem of the Baltic proper, which is of great interest from a management point of view.

## Introduction

**Fig. 1:**
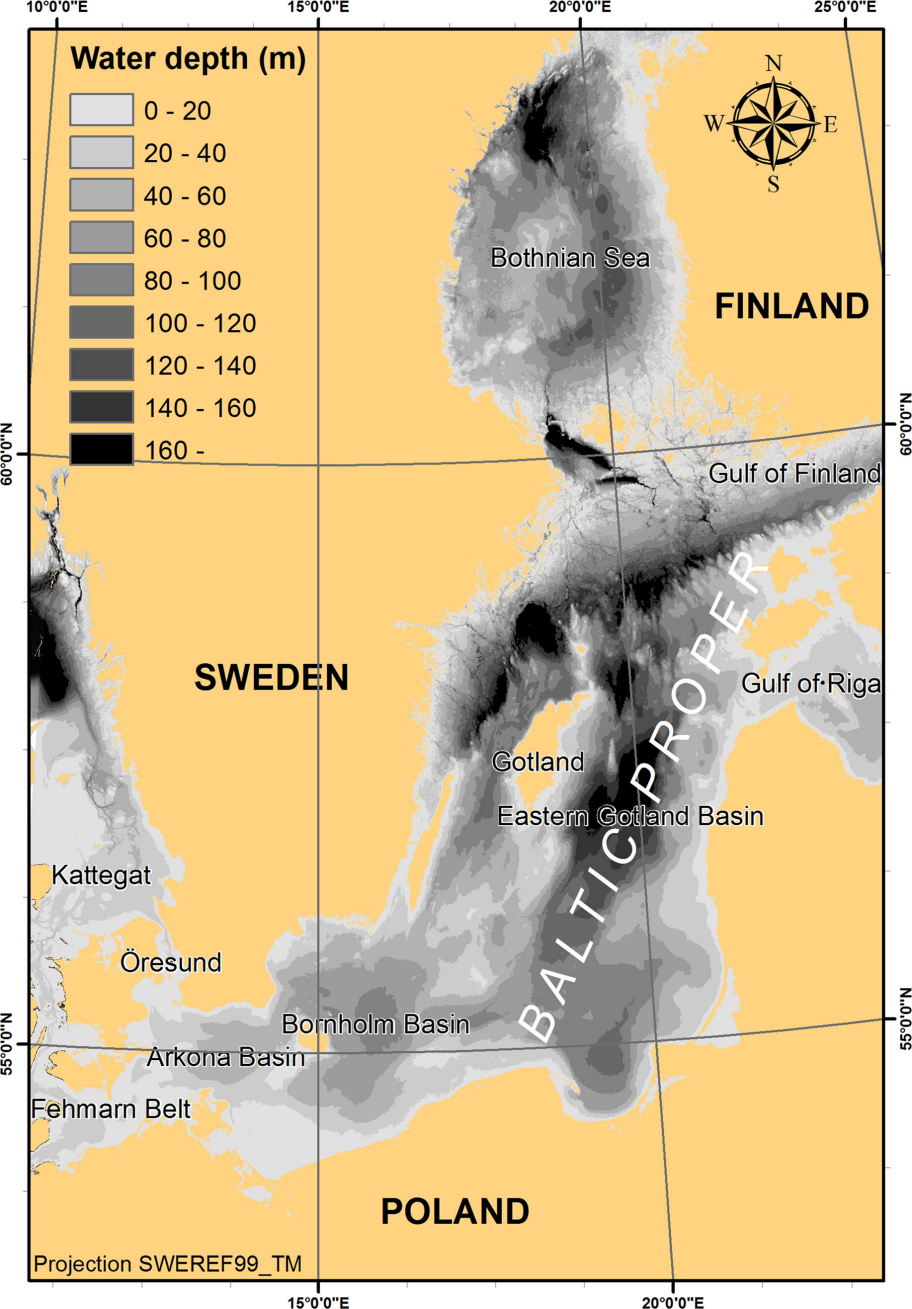
Map of the Baltic Sea

The Baltic Sea is a huge fjord-like water body with a large freshwater supply and deep basins beyond the shallow sills in the mouth. The surface salinity decreases from about seven in the Baltic proper to only 2–3 in the Bothnian Bay in the north (e.g. Leppäranta and Myrberg 2009). Freshwater flowing out from the Baltic Sea is stored in the surface layers of the Kattegat and the Belt Sea, outside the shallow entrance sills, see the map in Fig. 1. In this position the freshwater obstructs seawater from entering the Baltic proper across the shallow sills in the mouth and this leads to extensive recirculation of freshwater in connection with inflow of water to the Baltic Sea that occurs when the sea level stands higher outside than inside the entrance sills, see e.g. the detailed mechanistic model in Stigebrandt et al. (2015a). The deepwater salinity in the Baltic Sea decreases from 15 to 21 in the Bornholm Basin to 11–13 in the main basins of the Baltic proper (e.g. Leppäranta and Myrberg 2009). A strong halocline, usually starting at about 60 m depth in the main basins, obstructs mixing between the deepwater and the surface water. The residence time of water below the halocline varies much but is typically several years.

Phosphorus (P) concentrations have been observed in the Baltic proper since the 1930s although only sporadically until the 1960s. The earliest value found for the winter surface concentration is from 1958 when the horizontal mean phosphate [dissolved inorganic phosphorus (DIP)] concentration was about 0.18 mmol m^−3^ (Fig. 1 in Fonselius 1967), which should correspond to total P (TP) concentration of about 0.27 mmol m^−3^, using TP/DIP = 1.5 in the surface water in winter (L. Viktorsson, pers. communication). Fonselius and Valderama (2003) show that the DIP concentration at 100 m depth in the East Gotland Basin increased from less than 1 mmol m^−3^ in the middle of the 1930s to almost 3 at the end of the 1990s. The winter content of TP in the 60-m-thick surface layer of the Baltic proper for the period 1968–2010 was given in Fig 2 in Stigebrandt et al. (2014). The corresponding mean concentrations of TP can be estimated to about 0.8 in 1980, and 1.0 mmol m^−3^ in the period 2005–2010.

Inspired by lake modeling presented by Vollenweider (1969), an elementary time-dependent nutrient budget model was developed and applied to the Baltic Sea (Wulff and Stigebrandt 1989). From known external supplies and changed storage in the water column, the internal and external (by water export) sinks of nutrients were computed. Predictions were made of future nutrient states of the Baltic Sea for various nutrient loading scenarios. According to that model, the winter phosphorus concentration in the surface layer should decrease when the land-based external load decreases. However, contrary to the prediction, during the period 1980–2005 the winter surface P concentration increased by about 20% although the land-based external P supply was almost halved as shown by Stigebrandt et al. (2014) who concluded that the model by Wulff and Stigebrandt (1989) lacks a major P source, most probably located in anoxic bottoms. Therefore, an internal P source proportional to the area of anoxic bottoms was introduced in an extended time-dependent P budget model.

The amount of P annually stored in the bottom sediment (P sink) corresponds to a fraction of the P in the organic matter annually produced by biological production in the surface layer, see “Materials and methods” section below and e.g. Stigebrandt et al. (2014). Phosphorus may also come to the bottom sediment adsorbed to settling mineral particles (Fonselius 1967). Before the mid of the 1950s, probably little P was coming out from the sediment P sinks in the Baltic proper. However, when the deep waters for the first time in modern time (in the 1950s) became anoxic, huge amounts of phosphorus were released from the storage in the sediments (Fonselius 1967, 1970). It thus seems that the Baltic proper may provide long-term storage of P in the sediments if the sediments are overlain with oxic water. Sediment storage of P is further discussed in “Discussion” section.

**Fig. 2:**
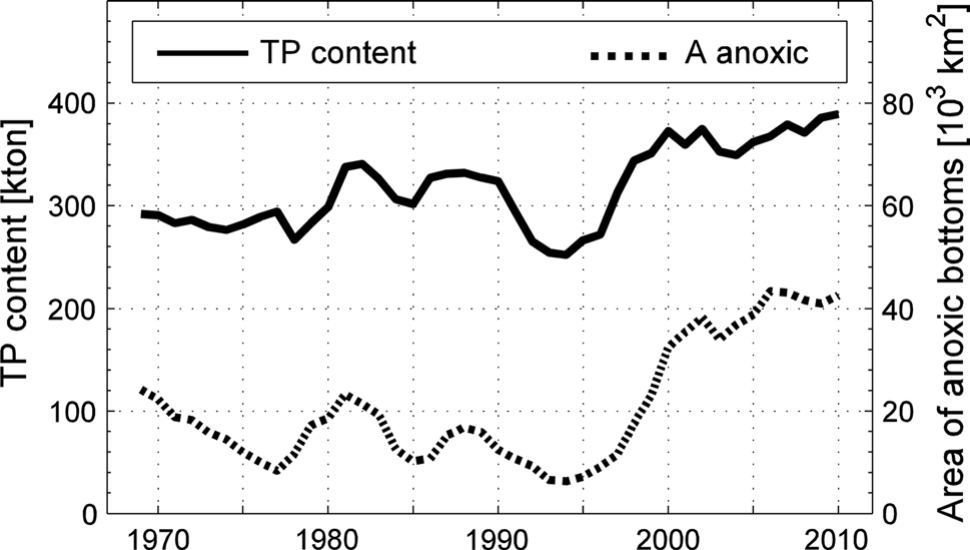
The content of P below 60 m (*solid line*) and the area of anoxic bottoms in the Baltic proper (*broken line*). (From Stigebrandt et al. 2014)

There is now massive evidence for oxygen control of the internal P source from anoxic sediments in the Baltic proper in its present state. Fonselius (1967) described how large amounts of P were released from the sediments in the East Gotland Basin when these were observed to become anoxic in the late 1950s. The model study of the Baltic proper P dynamics by Stigebrandt et al. (2014) builds on the hypothesis that oxygen controls the P release from sediments, and the temporal and spatial mean specific internal P source from anoxic bottoms was estimated to 2.3 g P m^−2^ year^−1^, which is supported by data on P release from sediments in the Bornholm Basin (3–8.6 g P m^−2^ year^−1^ during anoxic conditions and 0.8–1.5 g P m^−2^ year^−1^ during oxic conditions, these figures also contain P contributions from decomposition of fresh organic matter, about 0.7 g P m^−2^ year^−1^) presented in the same paper, and in situ observations of benthic fluxes from anoxic bottoms in the central Baltic Sea (4 ± 2 g m^−2^ year^−1^) reported by Viktorsson et al. (2013). Additional support was given by Stigebrandt et al. (2014) who showed a striking correlation between the area of anoxic bottoms and the P content in the water mass below 60 m depth in the Baltic proper, here reproduced in Fig. 2. By correlation between the annual variations of anoxic bottom area and the amount of phosphorus in the Baltic proper Conley et al. (2002) estimated that 2–5 g P m^−2^ year^−1^ were released in the expansion phase of anoxia. From nutrient budgets during stagnation periods in the deep water of the East Gotland Basin Gustafsson and Stigebrandt (2007) estimated single dose releases of about 3 g P m^−2^ when overlying water turns anoxic. Rosenberg et al. (2016) showed that the bottoms in the deep East Gotland Basin were oxidized in July 2015, a few months after inflow of new deepwater. Hall et al. (2017) observed that high efflux of P observed under anoxic conditions in the period 2008–2010 in the depth interval 170–210 m in the East Gotland Basin had turned to an influx of P in about 50% of the measurements in July 2015, when the oxygen concentration was 30–45 μM. Similar results with oxidation of the top of the bottom sediment and influx of P after oxygenation of the deepwater were obtained in the By Fjord (Stigebrandt et al. 2015b).

Sediment core data demonstrate that deepwater sediments in the Baltic proper have switched between oxygenated and anoxic episodes during the present several-thousand-years-long brackish water phase. Anoxic episodes might have been initiated by strong salinity stratification in the deepwater (Bianchi et al. 2000), which may cause prolonged residence time for the deepest deepwater during which oxygen may be exhausted even if the rate of supply of organic matter produced in the surface layer is quite small, like in the late 1950s as described by Fonselius (1967, 1970). The residence time of the deepest deepwater is then longer than the time it takes to consume the oxygen brought in by new deepwater. During anoxic episodes, there were simultaneous intense cyanobacterial blooms that likely were fueled by P released from anoxic sediments (Bianchi et al. 2000; Zillén and Conley 2010; Jilbert and Slomp 2013), an assumption in harmony with the parameterization of the internal source by Stigebrandt et al. (2014).

The finding that anoxic periods may terminate spontaneously demonstrates that the Baltic proper possesses a mechanism to restore itself from eutrophication, and a high-resolution study of sediment core data shows that terminations of anoxic periods have been rapid (Jilbert and Slomp 2013). This natural mechanism of restoration from eutrophication has not earlier caught much interest in the Baltic Sea literature. The crucial quality of a restoration mechanism should be that it may shut off the internal P source during a sufficiently long time TR during which the P content in the water column will decrease and approach a new equilibrium concentration determined by the P supply by external land-based and oceanic sources. Here TR is the inherent, system specific, time of restoration.

Efficient oxygenation of deep bottoms may occur if the well oxygenated surface layer expands vertically. This is believed to happen if the inflow of highly saline new deepwater from the entrance area is low in an extended period. The deepwater volume should then decrease because the volume of deepwater annually included by vertical mixing into the surface layer should exceed the volume of the annual refill of deepwater by inflow from the entrance area. It is suggested that a large-amplitude vertical expansion of the surface layer of duration comparable to TR provides a mechanism of restoration. Due to the huge variability of the salinity of new deepwater, see Stigebrandt et al. (2015a) and references therein, such periods are likely.

**Fig. 3:**
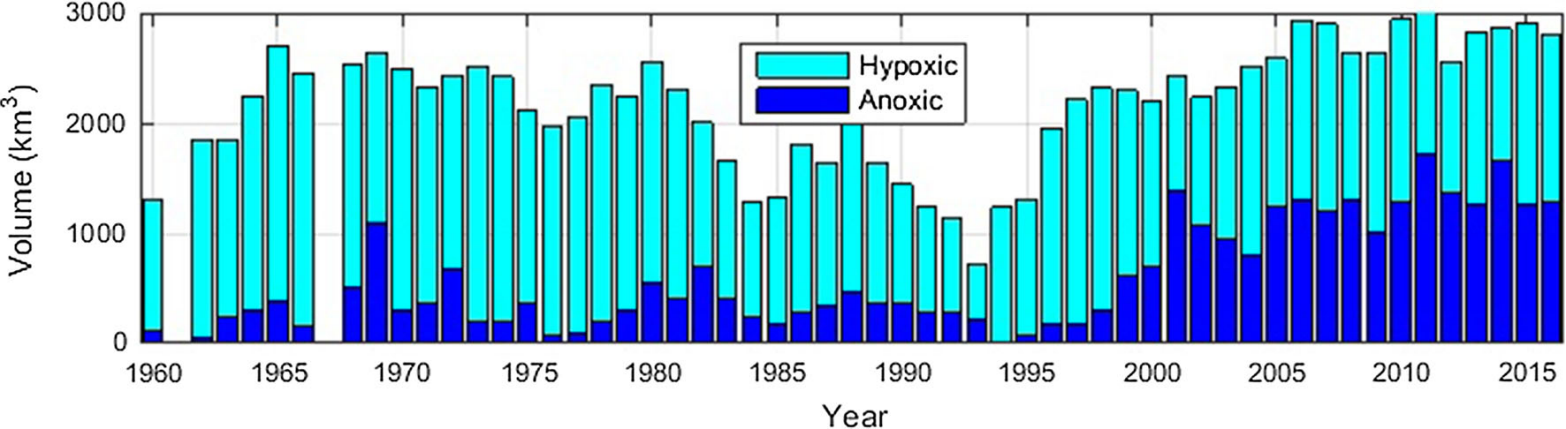
Volume of hypoxic (0 < O_2_ < 2 mL L^−1^) and anoxic (no oxygen) bottom water, observations obtained in the period August to October, in the Baltic proper, including Gulf of Riga and Gulf of Finland, from 1960 to 2015. Results from 1961 and 1967 were omitted due to lack of data from the deep basins. Redrawn from Hansson and Andersson (2017)

A long-lasting episode with a vertically expanded surface layer occurred from the middle of the 1980s to the end of 1992 during which the top of the halocline was lowered from its usual level at about 60 m to about 100 m depth and the P content decreased in the Baltic proper (Stigebrandt and Gustafsson 2007). Anoxic deepwater almost disappeared and the volume of hypoxic deepwater was halved (Fig. 3). However, this spontaneous restoration attempt by Nature did not last long enough to restore the Baltic proper. It was finished by a major inflow of new deepwater in 1993 whereby the halocline rose and the deepwater became more stratified. Inspired by the P reduction during this episode, Stigebrandt and Gustafsson (2007) proposed that sustained man-made oxygenation of the deepwater possibly could be used as a method to defeat eutrophication of the Baltic proper. Periods of deepwater renewal in the low-salinity inflow mode, recognized as periods with lowered halocline in the major basins in the Baltic proper, occurs rarely as can be seen from hydrographical data starting in the 1890s presented by e.g. Carstensen et al. (2014) and Stigebrandt (2001).

From the 1890s, when regular oxygen observations started, to the middle of the 1950s there were only small oxygen-free areas in the Baltic proper (Fonselius and Valderama 2003; Savchuk et al. 2008). Thereafter, anoxic bottoms expanded quickly, probably promoted by increased respiration linked to increased land-based external supply of P (Fonselius 1970; Carstensen et al. 2014). However, the strongly increased vertical stratification in the deepwater, induced by the extremely large and salty inflow of new deepwater in 1951, led to prolonged residence time of the deeper deepwater which caused expansion of anoxia and very large internal supply of P released from anoxic bottoms (Fonselius 1967). Decreased oxygen solubility due to warming of the deepwater of the Baltic Sea during the last century (e.g. Fonselius and Valderama 2003) may have worsened hypoxia and anoxia, e.g. Carstensen et al. (2014).

Due to increased sewage treatment and improved agricultural practice, the land-based external P supply to the Baltic proper decreased by about 50% from the peak in the 1980s to present time. Based on numbers given by Gustafsson et al. (2012), Stigebrandt et al. (2014) used an external P supply of 60 000 and 35 000 tonnes year^−1^ in 1980 and 2005, respectively for the Baltic proper. Despite this large reduction, the winter P content in the water column, both in the 60 m deep surface layer and in the deepwater, increased by about 20% during the same period (Stigebrandt et al. 2014). The increasing P content in the water column of the Baltic proper can be explained by an increasing internal P source, increasing from ca 46 000 tonnes year^−1^ in 1980 to 92 000 in 2005 (Stigebrandt et al. 2014), which obviously more than well compensated for the decreasing land-based external P supply. The increased eutrophication (Fig. 2) and the increased volume of anoxic water occurring after the ending of the spontaneous restoration attempt in 1993 (Fig. 3) can thus be explained by the increasing internal source, which is explained by the increasing area of anoxic bottoms which is explained by a lack of oxygenation of deep bottoms in combination with high supply of organic matter from the eutrophic surface layer.

This paper is organized as follows. The P budget model of the Baltic proper is described in “Materials and methods” section. A formula is derived in “The equilibrium winter surface P concentration” section for the equilibrium (steady-state) winter surface layer concentration of P as function of the total P supply, which equals the sum of the land-based and oceanic external and the internal P supplies. In “The adjustment time to changed total P supply” section it is shown that the temporal response of the winter surface layer concentration to a changed total P supply is described by an exponential function which defines the inherent restoration time of the Baltic proper. This is followed by the presentation and discussion of model results in “Results” section. Prerequisites for restoration of the Baltic proper by man-made oxygenation of the deepwater are discussed in “Discussion” section and the paper is brought to an end by some concluding remarks in “Concluding remarks” section.

## Materials and methods

**Fig. 4:**
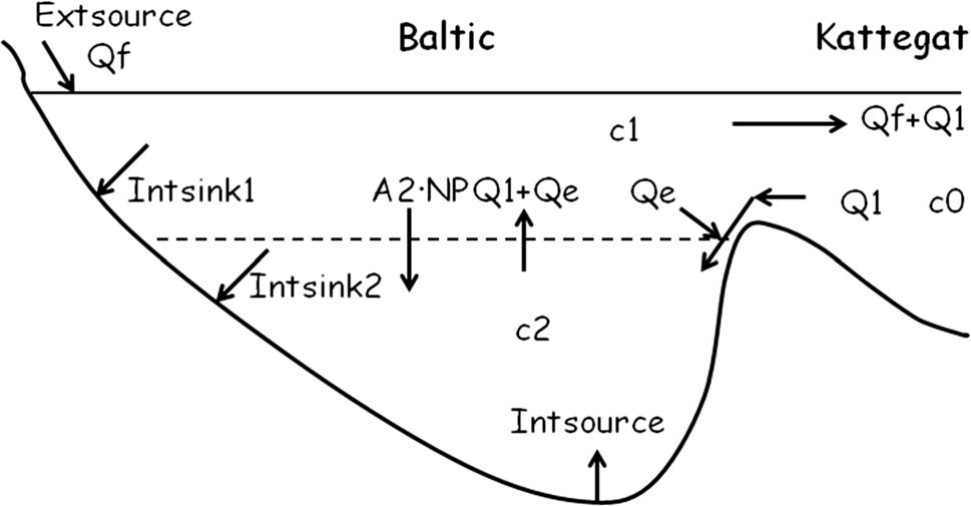
Phosphorus model of a two-layered Baltic Sea with winter concentrations *c*_1_ and *c*_2_, in the upper and lower layers, respectively (Stigebrandt et al. 2014). The *stippled line* indicates the horizontal surface of area *A*_2_ separating the upper and lower layers (the halocline). The P concentration, *c*_0_, in Kattegat, outside the entrance sills is further discussed below Eq. (1). *Q*_f_ is the rate of freshwater supply, *Q*_1_ the rate of inflow of new deepwater from Kattegat and *Q*_*e*_ the flow rate of surface water that is entrained into the inflowing new deepwater. The vertical exchange of P between the layers is due to (*downward*) export of new production *NP* and surface water mixed into the new deepwater from Kattegat and (*upward*) transport of lower layer water into the surface layer due to entrainment in winter at the mean rate *Q*_1 +_ *Q*_e_. Internal sources and sinks associated to the seabed are also marked

In the present paper, the time constant of the adjustment process following a changed P supply is derived. The steady-state (equilibrium) winter surface water P concentration in the Baltic proper that would be attained at the end of a restoration effort, is also derived. The salinity stratified Baltic proper has two layers (Fig. 4). Each autumn and winter the upper layer is vertically mixed down to the halocline whereby water from the lower layer is entrained into the surface layer. The lower layer is vertically stratified. For the physics of the Baltic Sea see e.g. Stigebrandt (2001) and Leppäranta and Myrberg (2009). The time-dependent phosphorus model is a mass balance model that considers sources and sinks, that all can be represented by fluxes through the external boundaries of the water mass, and storage changes in the water mass (Fig. 4). For an explanation of symbols used, see Table 1.

**Table 1:** List of symbols

*a*	Integration constant
*A* [km^2^]	Horizontal surface area of the Baltic proper
*A*_*2*_ [km^2^]	Horizontal surface separating the two layers, c.f. Fig. 4
*A*_anox_ [km^2^]	Area of anoxic bottoms
*A*_df_ [km^2^]	Area of defaunated bottoms (before oxygenation starts)
*b*	Integration constant
$\bar{c}$ [mmol P m^−3^]	Mean winter P concentration in the volume V
*c*_1_ [mmol P m^−3^]	Winter P concentration in the surface layer
$\bar{c}_{1}$ [mmol P m^−3^]	Annual mean P concentration in the surface layer
*c*_1*e*_ [mmol P m^−3^]	Equilibrium winter surface P concentration
*c*_1*i*_ [mmol P m^−3^]	Initial winter surface P concentration (at the start of restoration)
*c*_2_ [mmol P m^−3^]	Winter P concentration in the lower layer
*c*_0_ [mmol P m^−3^]	Time mean P concentration of inflowing new deepwater from Kattegat
Extsource [tonnes P year^−1^]	External land-based P source
*fs* [g P m^−2^ year^−1^]	Temporal and spatial mean specific DIP flux from anoxic bottoms
Intsink [tonnes P year^−1^]	Total Internal sink of P, defined by Eq. (2), c.f. Fig. 4
Intsource [tonnes P year^−1^]	Internal P source from anoxic bottoms
NP [tonnes P year^−1^]	Net production
*Q*_1_ [km^3^ year^−1^]	Rate of inflow of new deepwater from Kattegat, c.f. Fig. 4
*Q*_1_ *c*_0_ [tonnes P year^−1^]	External oceanic P source (P inflow from Kattegat)
*Q*_e_ [km^3^ year^−1^]	Rate of entrained surface water into the new deepwater, c.f. Fig. 4
*Q*_f_ [km^3^ year^−1^]	Rate of freshwater supply, c.f. Fig. 4
*t* [year]	Time
*T* [year]	Time constant for the adjustment process
Totsource [tonnes P year^−1^]	Equals Extsource + *Q*_1_ *c*_0_ + Intsource
TR [year]	Restoration time defined as TR = 3*T*
TRVF [km^3^ year^−1^]	Total Removal Volume Flux
*V* [km^3^]	Total Volume of Baltic proper
*V*_1_ (*V*_*2*_) [km^3^]	Volume of upper (lower) layer
*v* [m year^−1^]	Apparent settling velocity
*x* [mmol P m^−3^]	Substitution variable used for solving Eq. (7)
*α*	Fraction (0 ≤ *α*≤1) of P in benthos grazed by demersal fish that emanates from SOM
*β*	Fraction (0 ≤ *β*≤1) of P in demersal fish that is excreted
$\gamma = \bar{c}_{1} /c_{ 1}$	Ratio *γ* equals 0.8 (Wulff and Stigebrandt 1989)
*δ*	Fraction (0 ≤ *δ*≤1) of earlier defaunated bottoms that are colonized after long-term natural or artificial oxygenation

Following Stigebrandt et al. (2014), the time-dependent equation (time resolution 1 year) for the total content of phosphorus in the water column of the Baltic proper reads

1 $$ V\frac{{{\text{d}}\bar{c}}}{{{\text{d}}t}} = {\text{Extsource}} + {\text{Intsource}} + Q_{1} c_{0} - (Q_{f} + Q_{1} )\bar{c}_{1} - {\text{Intsink}} $$

here *V* is the volume of the Baltic proper and $\bar{c}$ is the volume mean winter concentration of phosphorus so that *V* $\bar{c}$ is the total winter content of P in the water column. Extsource is the land-based external P source; *Q*_*f*_ is the rate of freshwater supply; *Q*_*1*_ is the rate of inflow of new deepwater from Kattegat c.f. Fig. 4. $\bar{c}_{1}$ is the annual mean concentration in the surface layer and *c*_0_ is the time mean concentration of phosphorus in the water flowing into the Baltic from the entrance area. *Q*_1_ *c*_0_ is the oceanic source of P, i.e. import from the Kattegat area, while *(Q*_f_ + *Q*_1_ *)* $\bar{c}_{1}$ is the export of P. It is assumed that $\bar{c}_{1} = \gamma c_{1},$ where *c*_1_ is the winter concentration of phosphorus in the surface layer (Wulff and Stigebrandt 1989). The internal source term, Intsource, constitutes the very important difference between the improved model described by Eq. (1) and the old model by Wulff and Stigebrandt (1989) where it is lacking. Using data for 1980 and 2005, Stigebrandt et al. (2014) found that Intsource = *f*s · *A*_anox_ where *f*s = 2.3 g P m^−2^ year^−1^ is the temporal and spatial mean-specific DIP flux from anoxic bottoms and *A*_anox_ is the area of anoxic bottoms. Please remember that the internal dynamics that regulate the exchange between the layers are not invoked in the P model.

The deepwater in the 100-m deep Bornholm Basin in the southern Baltic proper switches since the 1960s between oxic and anoxic conditions. Observations from this basin confirm that the internal P source is turned on only during anoxic conditions and turned off again when the bottom water is oxygenated (Stigebrandt et al. 2014). Please note that in that study it was possible to differentiate between the P flux from internal sources and the P flux from decomposition of fresh organic matter. Observations in the Baltic proper show that the top layer of the sediment was rapidly oxygenated by the recent major deepwater inflow (Rosenberg et al. 2016). Additional examples of oxygen control of the internal P source from anoxic bottoms were presented in the introductory section. Long-term effects of oxygenation on P fluxes were discussed by Stigebrandt et al. (2015a). P storage in sediments is further discussed in “Discussion” section.

The internal sink, Intsink = Intsink_1_ + Intsink_2_ (c.f. Fig 4), can be written as (Wulff and Stigebrandt 1989)

2 $$ {\text{Intsink}} = c_{1} vA. $$

here *v* (m year^−1^) is the so-called apparent settling velocity and *A* is the surface area of the Baltic proper. The annual removal rate of phosphorus from the surface water to internal sinks is related to the biological production and is thus assumed to be proportional to the upper layer winter concentration *c*_1_ as explained by Wulff and Stigebrandt (1989). The external sink by export to Kattegat is also proportional to *c*_1_ as discussed above. Thus, all sinks are proportional to the winter surface water concentration *c*_*1*_.

Using Eq. (2), and writing Totsource = Extsource + Intsource + *Q*_1_ *c*_0_, and introducing the quantity Total Removal Volume Flux to the sinks, defined by TRVF = *νA* + *γ* (*Q*_f_ + *Q*_1_), the winter surface concentration *c*_1_ (mmol P m^−3^) can be written as follows (from Eq. 1):

3 $$ c_{1} = \frac{{{\text{Totsource}} - V\frac{{{\text{d}}\bar{c}}}{{{\text{d}}t}}}}{\text{TRVF}} $$

The winter surface concentration *c*_1_ in the Baltic proper is thus proportional to the total supply of phosphorus minus the rate of change of P stored in the water column. It can be seen that *c*_1_ is inversely proportional to TRVF, the total removal volume flux to the sinks.

The numerical value of the denominator TRVF in Eq. (3) will be determined here. In 1980, the winter concentration *c*_*1*_ of phosphorus (TP) in the surface layer was 0.8 mmol P m^−3^, Extsource = 60 000, Intsource = 46 000, $V\frac{{{\text{d}}\bar{c}}}{{{\text{d}}t}} = 5000$ and $Q_{1} c_{0} = 1 1\,000 \, \left( {{\text{tonnes P year}}^{ - 1} } \right),$ see Table 2. The estimated oceanic import *Q*_1_ *c*_0_ is similar to other estimates as discussed in Stigebrandt et al. (2014). Inserting this in Eq. (3) one finds that TRVF = *vA* + γ (Q_f_ + Q_1_) = 4520 km^3^ year^−1^. With *Q*_1_ ≈ *Q*_f_ ≈ 450 km^3^ year^−1^ (e.g. Stigebrandt et al. 2014) and γ = 0.8 (Wulff and Stigebrandt 1989), the flushing term *γ* (*Q*_f_ + *Q*_1_) equals 720 km^3^ year^−1^. The value of the internal sink term *vA* thus equals 3800 km^3^ year^−1^. According to the present model, the sink by export to Kattegat accounts for 16% (720/4520) while the internal sink accounts for 84% (3800/4520) of the total sink. The internal phosphorus sink, which in the present case also includes P exported to the Bothnian Sea (Stigebrandt et al. 2014), is thus 5.3 times greater than the export sink to Kattegat. Within the frame of the budget model, variations of the physical circulation system are not considered.

**Table 2:** Column 2 shows parameter values valid for 1980 for evaluation of TRVF Adopted from Stigebrandt et al. 2014)

*C*_1_ (mmol P m^−3^)	0.8	Surface winter concentration, observed
Extsource (tonnes P year^−1^)	60 000	External land-based source
Intsource (tonnes P year^−1^)	46 000	Internal source
*V(d* $\bar{c}$ */*d*t)* (tonnes P year^−1^)	5000	Annual storage change
*Q*_1_ *c*_0_ (tonnes P year^−1^)	11 000	External oceanic source
*Q*_f_ (km^3^ year^−1^)	450	Freshwater supply
*Q*_1_ (km^3^ year^−1^)	450	Inflow from Kattegat
*γ*	0.8	Concentration factor

The error in *c*_1_ as determined from the total P content in the upper layer is estimated to be ±0.05 mmol m^−3^, c.f. Fig. 2 in Stigebrandt et al. (2014). The error in TRVF is of the same magnitude as the error in Totsource—$V\frac{{{\text{d}}\bar{c}}}{{{\text{d}}t}}$. The errors in both Extsource and $V\frac{{{\text{d}}\bar{c}}}{{{\text{d}}t}}$ are discussed in Stigebrandt et al. (2014). Intsource was estimated in Stigebrandt et al. (2014) using data from 1980 and 2005 and the error should be of the same magnitude as the errors in the other source terms. Assume that the error in Totsource—$V\frac{{{\text{d}}\bar{c}}}{{{\text{d}}t}}$ is ±20%. Then TRVF should be 4520 ± 950 km^3^ year^−1^.

With *A* = 250 000 km^2^, one finds that the apparent removal rate *v* ≈ 15 m year^−1^, c.f. Equation (2). This is comparable to 14–15 m usually found in lakes (Reckhov and Chapra 1983) but it is twice the value estimated by Wulff and Stigebrandt (1989). The lower value found by Wulff and Stigebrandt (1989) should be due to their neglection of the internal source, an explanation also suggested by them. With *c*_1_ = 0.8 mmol P m^−3^, Intsink amounts to about 93 000 tonnes year^−1^ which is quite close to the value (93 750 tonnes year^−1^) estimated in Stigebrandt et al. (2014).

Below, two additional results of the phosphorus model are derived. The first result is a formula for the equilibrium concentration *c*_1e_, which is the steady-state concentration that should occur after a sufficiently long time of constant P supply. The second result is the response time of *c*_1_ to changes of the total P supply. The response time is system-dependent and determines the inherent restoration time of a system.

### The equilibrium winter surface P concentration

**Fig. 5:**
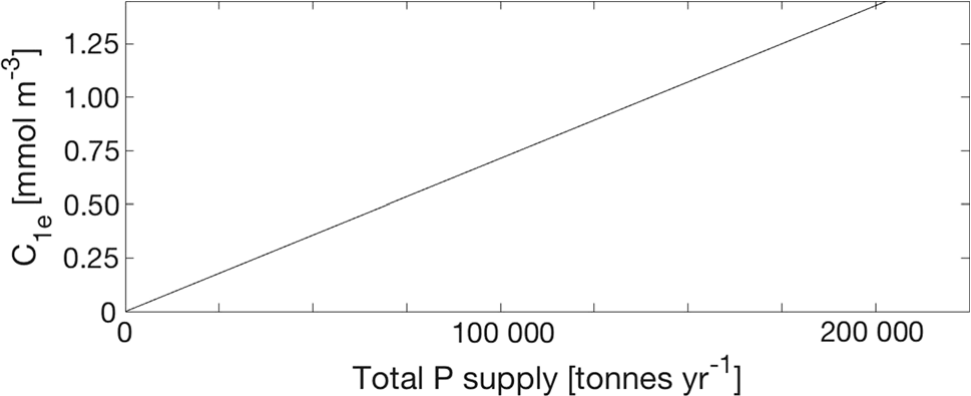
The equilibrium winter surface concentration *c*_1e_ (TP) versus the total P supply to the Baltic proper computed using Eq. (4)

For steady-state situations, the total sink = *c*_1e_·TRVF equals the total source, Totsource, and the storage of P in the water column and the total supply do not change. For this situation, Eq. (3) shows that the equilibrium concentration in the upper layer *c*_1e_ equals

4 $$ c_{{1{\text{e}}}} = \frac{\text{Totsource}}{\text{TRVF}}. $$

here TRVF, the total removal volume flux, equals 4520 km^3^ year^−1^ for the Baltic proper as estimated above. This result for the equilibrium concentration, Eq. (4), is also displayed in Fig. 5. It should be extremely interesting from e.g. a management point of view. It is discussed in “The equilibrium solution” section.

### The adjustment time to changed total P supply

The adjustment of the surface water winter concentration c_1_ towards the new equilibrium concentration c_1e_ when the total P source Totsource is changed abruptly is estimated using Eq. (3). This equation contains the mean concentration in the water column in winter, $\bar{c}$, which is defined by

5 $$ V\bar{c} = V_{1} c_{1} + V_{2} c_{2}. $$

here *V* = *V*_*1*_ + *V*_*2*_ and (*V*_*1*_ *,V*_*2*_) are the volumes and (*c*_*1*_ *, c*_*2*_) the winter P concentrations of the two layers. For the understanding and description of the adjustment process, it is valuable to find analytical solutions if possible. In the following, two cases are studied. In Case 1, the lower layer has disappeared and there is only a surface layer. It is assumed that this case might occur during a period with low salinity mode of water renewal as discussed in “Introduction” section. It reminds of the case believed to occur when the Baltic Sea restores itself spontaneously as argued in “Introduction” section. In Case 2, the stratification is as usual, with a halocline at 60-m depth and the lower layer is kept oxygenated by some unspecified method.

#### Case 1

In this case, the lower layer has disappeared, i.e. *V*_*2*_ = 0, due to only small inflows of new dense water. With *V*_2_ = 0 it follows from Eq. (5) that *V* = *V*_*1*_ and $\bar{c} = c_{1}$. Equation (3) is then written.

6 $$ \frac{{{\text{d}}c_{1} }}{{{\text{d}}t}} = \frac{{{\text{Totsource}} - c_{1} \cdot {\text{TRVF}}}}{V}. $$

Using Eq. (4), Eq. (6) can be written.

7 $$ \frac{{{\text{d}}c_{1} }}{{{\text{d}}t}} = \frac{\text{TRVF}}{V}(c_{{1{\text{e}}}} - c_{1} ). $$

Make use of the following substitution of variables

8 $$ x = c_{1} - c_{{1{\text{e}}}}. $$

One then obtains

9 $$ \frac{{{\text{d}}x}}{{{\text{d}}t}} = - \frac{\text{TRVF}}{V}x. $$

Equation (9) has the following solution

10 $$ x = a \cdot e^{{ - \frac{t}{T}}} + b. $$

here *T* is the time constant for the adjustment process. *T* is defined by

11 $$ T = \frac{V}{\text{TRVF}}. $$

Change variables again using Eq. (8).

12 $$ c_{1} - c_{{1{\text{e}}}} = a \cdot e^{{ - \frac{t}{T}}} + b. $$

The temporal boundary conditions are applied to determine the two integration constants, *a* and *b*. For long times (*t* → ∞), we expect that *c*_1_ attains the equilibrium concentration *c*_1e_. This gives *b* = 0. At *t* = 0, i.e. at the start of the change of supply, *c*_1_ equals the initial concentration *c*_1i_. This gives *a* = *c*_1i_ − *c*_1e_.

Finally, insertion of the integration constants *a* and *b* gives the solution of Eq. (6)

13 $$ c_{1} = c_{{1{\text{e}}}} + (c_{{1{\text{i}}}} - c_{{1{\text{e}}}} )e^{{ - \frac{t}{T}}}. $$

with *V* = 14 780 km^3^ (Stigebrandt 1987) and TRVF = 4520 km^3^ year^−1^ (given above in “Materials and methods” section), one obtains *T* = 3.27 year.

**Fig. 6:**
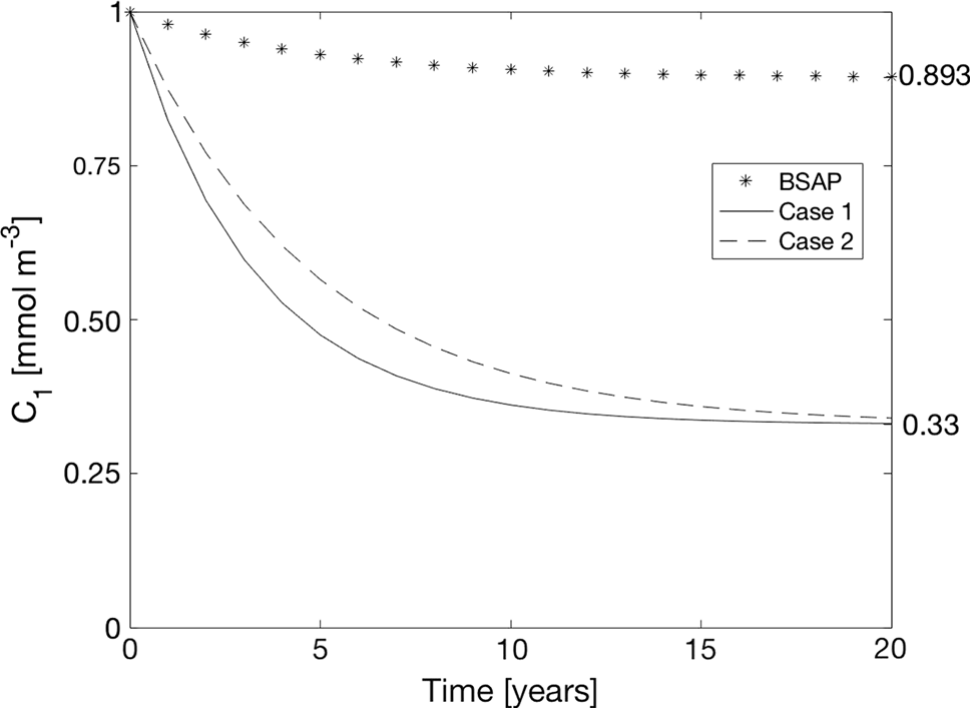
Adjustment of the winter surface layer concentration *c*_1_ towards the equilibrium concentration c_1e_ for two cases described in “The adjustment time to changed total P supply” section and the “BSAP case” described in “The equilibrium solution” and “Response time of the winter surface concentration of P to changed total supply of P” sections

The inherent restoration time TR is here defined so that 5% of the difference between the initial and the equilibrium concentrations remain (Fig. 6). This gives TR = 3*T*. In Case 1, restoration may thus be performed in about 10 years.

#### Case 2

In this case, the 2-layer stratification is maintained and the lower layer is kept oxygenated. This should be possible to achieve by man-made and possibly also by natural oxygenation. During the period 1970–2010, there were about equal amounts of P in the upper and lower layers, i.e. *V*_1_ *c*_1_ = *V*_2_ *c*_2_, cf. Fig. 2 in Stigebrandt et al. (2014). Assuming that this is true for Case 2, one may replace *V* $\bar{c}$ by 2*Vc*_*1*_. Equation (3) is then rewritten.

14 $$ \frac{{{\text{d}}c_{1} }}{{{\text{d}}t}} = \frac{{{\text{Totsource}} - c_{1} \cdot {\text{TRVF}}}}{{2V_{1} }}. $$

The only difference between Eq. (6) and Eq. (14) is the value of the denominator on the right side; *V* in Eq. (6) has been replaced by 2*V*_1_ in Eq. (14). The solution of Case 1 in Eq. (13) is valid also for Eq. (14) if the value of *T* in Eq. (11) is replaced by *T* = 2*V*_*1*_/TRVF. With *V*_*1*_ = 10 790 km^3^ (Stigebrandt 1987), one obtains *T* = 4.77 year. The restoration time in Case 2 is thus about 14 years, c.f. Fig. 6. The reason why the response is somewhat slower in Case 2 than in Case 1 is because *c*_*1*_ in Case 2 is lower than the vertical mean concentration, which makes the sink rates smaller in Case 2 than in Case 1.

When solving Eqs. (6) and (14), it was assumed that Totsource, which equals the land-based external supply plus the ocean supply, was constant. If Totsource changes with time during the restoration process, *c*_1e_ will change according to Eq. (4). However, solutions still follow Eq. (13) but with values of *c*_1i_ and *c*_1e_ that are changing step by step as Totsource changes.

## Results

### The equilibrium solution

The equilibrium (steady-state) solution for the winter surface concentration *c*_*1e*_ for different values of the total P supply is given by Eq. (4) and drawn in Fig. 5. The realism of the predicted equilibrium concentration for different values of the total P supply can be tested by comparison with the observed TP concentration in the winter surface water presented in “Introduction” section. If the loading is increasing, the observed concentration in a certain year should be lower than the equilibrium concentration based on the total P supply that year, and vice versa. Taking this into account, it can be concluded that the model is quite realistic (Table 3) and the model result supports that the equilibrium winter surface concentration is proportional to the Total P supply Totsource as defined by the model. This is a very strong support for the idea that anoxic bottoms in the Baltic proper act as long-term P sources.

**Table 3:** The observed TP concentration *c*_1o_ (column 3) in the winter surface water in the Baltic proper and the modeled, equilibrium concentration from Eq. (4), *c*_1e_ (column 4). The observed concentrations are from “Introduction” section of the present paper. The Total P loading Totsource (column 2) equals the sum of land-based and ocean-based external sources and the internal source, is obtained from numbers given by Stigebrandt et al. (2014)

Year	Totsource (tonnes P year^−1^)	*c*_1o_ (mmol P m^−3^)	*c*_1e_ (mmol P m^−3^)
1958	45 000	0.27	0.32
1980	117 000	0.80	0.83
2010	140 000	1.00	1.00

The equilibrium solution is of great interest to management. The potential of changing the oceanic source, *Q*_1_ *c*_0_, appears very small so this is not discussed here. The potential of decreasing the land-based external source is probably about 15 000 tonnes P year^−1^ which is also the long-term goal of HELCOM Baltic Sea Action Plan (HELCOM Ministerial Meeting 2007). Decreasing the external source by 15 000 tonnes P year^−1^, the “BSAP case”, would decrease Totsource by about 11%, from 140 000 (obtained from Eq. (4) using *c*_1e_ = 1 mmol m^−3^) to 125 000 tonnes P year^−1^. According to Eq. (4), this would decrease *c*_1e_ by about 11%. Such a small change might be hard to verify mainly due to the large variability of the external sources, the internal source and the circulation system.

Since the internal source of P emanates from anoxic bottoms, sustained oxygenation of the deep bottoms of the Baltic proper would shut off the internal source (“Introduction” section), Totsource should then decrease from 140 000 to 46 000 tonnes year^−1^, or less, depending on the magnitude of the external P supply at the time for restoration. The equilibrium winter surface concentration *c*_1e_ after restoration should then be 0.33 mmol P m^−3^ or lower (c.f. Fig. 5), which is a reduction by at least 67% as compared to the contemporary concentration of about 1 mmol P m^−3^.

The net (export) production of organic matter on an annual basis *NP*, c.f. Fig. 4, is P limited (e.g. Conley et al. 2002; Boesch et al. 2006; Olofsson et al. 2016). The rate of loading of the deepwater with fresh organic matter is thus proportional to the winter surface concentration *c*_1_. This means that if restoration reduces *c*_1_ by e.g. *R*%, the deepwater oxygen demand due to supply of fresh organic matter is reduced by *R*%. Thus, during the restoration operation, the deepwater oxygen demand is strongly reduced. One may then expect that the natural vertical circulation of the Baltic proper should be able to keep the deepwater oxygenated after restoration in the same manner as it did for a long period ending in the 1950s, e.g. Fonselius and Valderama (2003) and Savchuk et al. (2008).

### Response time of the winter surface concentration of P to changed total supply of P

In “The adjustment time to changed total P supply” section the temporal response of the winter surface concentration c_1_ to shutting off the internal P source is computed. The time-dependent equation for the winter surface concentration *c*_1_ is solved for two cases. In Case 1, it is assumed that the lower layer does not exist so that the upper layer fills the whole Baltic proper. This is like the case that is expected to occur in periods when the Baltic proper restores itself as discussed in “Introduction” section. It is found that the temporal response to shutting off the internal P source is described by an exponential function with the e-folding time *T* equal to about 3.5 years. In Case 2, it is assumed that the lower layer exists all the time and that the halocline is at 60 m depth. The lower layer is in some way kept oxygenated so that the internal P source is shut off. The equation has the same solution for this case, except for the time constant T that is a little longer, about 4.4 years. The restoration time TR, defined as TR = 3*T*, would be in the range 10–14 years depending on the vertical stratification, c.f. the graphical presentation in Fig. 6. After TR years the eutrophication surplus should be 0.05 (*c*_1t_–*c*_1e_). The rapid exponential decay with TR of the order of one decade, predicted by the present analysis, fits nicely with the observation in sediments of rapid termination of anoxic periods shown by Jilbert and Slomp (2013). During the revision of the present paper, it became known that Katsev (2017) derived the e-folding time of response and the steady-state P concentration, the same quantities as those derived in the present paper, using a one-box model that includes the recycling efficiency of P in the sediment with application to large lakes.

It should be noted that the response time to a change in the total supply of P derived in “The adjustment time to changed total P supply” section should be valid for all kinds of changes of the P supply, thus even for changes of the external supply. The “BSAP case” is shown in Fig. 6 but with the land-based external source momentarily reduced with 15 000 tonnes P year^−1^. This case demonstrates that not even a full implementation of the Baltic Sea Action Plan would solve the problem of eutrophication of the Baltic proper if the huge internal source persists.

## Discussion

It was shown in “Results” section that turning off the internal source would reduce the total source by about 70% which in 10–15 years would reduce the winter surface P concentration by about 70%. But is it possible to achieve such an enormous improvement of the state of the Baltic proper? Some important aspects are discussed below.

Stigebrandt and Gustafsson (2007) suggested that oxygen for deepwater oxygenation should be taken from the oxygen saturated so-called winter water, resting above the permanent halocline with oxygen content greater than 10 g O_2_ m^−3^. They estimated that an oxygen flux of 3 × 10^9^ kg year^−1^ would be needed to keep the deepwater oxygenated and suggested that floating wind mills equipped with pumps might be used to transport winter water into the deepwater where it should be mixed with the ambient water. A similar system, powered by the electrical grid, was used in the By Fjord experiment where 2 m^3^ s^−1^ of surface water was pumped into the deepwater and released through horizontal jets to obtain strong instantaneous mixing without stirring up bottom sediments. The pumping created a large downward vertical motion in the basin (about 1 m day^−1^) because the buoyant plumes entrain large amounts of deepwater that are lifted upwards (c.f. Fig. 2 in Stigebrandt et al. 2015b). A theoretical pumping experiment showed that the Bornholm Basin can be kept well oxygenated by pumping 1000 m^3^ s^−1^ of winter water into the deepwater (Stigebrandt et al. 2015a). In both the By Fjord experiment and the theoretical pumping experiment in the Bornholm Basin, increased mixing by the pumping increased quite much the rates of water exchange and the accompanying oxygen supply to the deep basins. The exact oxygen need of a complete restoration of the Baltic proper, the optimal design of pumps and their geographical localization and the most efficient power supply remain to be investigated.

When deepwater oxygenation starts, there is an initial oxygen debt composed by hydrogen sulfide and ammonium dissolved in the water that must be paid. In the autumn of 2013, the amount of oxygen needed to oxidize all hydrogen sulfide and ammonium present in the anoxic deepwater of the Baltic proper were about 2 × 10^9^ and 0.5 × 10^9^ kg, respectively, as estimated from hypsographic data and data on hydrogen sulfide and ammonia (the SHARK database, hosted by SMHI). The hydrogen sulfide and ammonium oxygen debts plus a debt due to reduced substances in a top layer of the sediment were included in the model of the Bornholm Basin by Stigebrandt et al. (2015a). It was found that the sediment debt was significant but the effect it had was small compared to other processes. Natural oxygenation events due to large inflows of new deepwater to the Baltic proper in the period 1960–1993 were relatively successful in oxygenating the deep bottoms although Fonselius (1970) stated that a substantial part of the oxygen in new deepwater was used to oxidize the sulfide and he concluded that the Baltic proper can no longer in a natural way recover from what he called “the hydrogen sulfide shocks”. The very large recent inflow in December 2014 (Mohrholz et al. 2015) failed to oxygenate all the deepwater of the Baltic proper. This was expected since the volume of anoxic water increased by a factor of about 7, from typically 200 km^3^ in the period 1960–1998 to typically 1400 km^3^ after 2001 (c.f. Fig. 3). A preliminary estimate suggests that an initial oxygen debt will prolong the time it takes to restore the Baltic proper, by about 1 year if the debt is about 3 × 10^9^ kg oxygen.

Objections against artificial oxygenation of the deepwater of the Baltic proper have been raised because of expected negative ecological effects, described in e.g. Conley et al. (2009) and in the brief review in Stigebrandt et al. (2014). All suggested negative as well as positive effects of oxygenation should be analyzed in an Environmental Impact Assessment (EIA) before a decision to restore the Baltic proper is taken. Work that can be included in an EIA has already been published. In the By Fjord experiment (Stigebrandt et al. 2015b), it was shown that oxygenation of the sediment did not increase the fluxes of organic and inorganic toxins from the earlier anoxic sediments. Other effects of oxygenation are discussed in the paper by Stigebrandt et al. (2014). A model investigation of the effect of man-made oxygenation on the cod recruitment in Bornholm Basin shows that oxygenation of the deep waters in the Bornholm Basin should improve the hydrographical conditions required for successful cod recruitment (Stigebrandt et al. 2015a). This is measured by the so-called cod reproduction volume (CRV defined by; *S* > 11, O_*2*_ > 2 mL L^−1^). The model computations show that in years when the CRV was small in the Bornholm Basin under natural conditions, oxygenation would have helped to increase the CRV substantially. Keeping the deepwater oxygenated will permit colonization of the deep bottoms of the basin which will increase the food supply to e.g. cod. It will also stop leakage of phosphorus from the earlier periodically anoxic bottoms, which would be the very reason for undertaking oxygenation. A restoration system also has legal and fiscal aspects that require negotiations between the Baltic Sea states.

Since the equilibrium model, forced by the total P supply, describes the evolution of the Baltic proper from the oligotrophic state in the 1950s to the present (mildly) eutrophic state quite well (Table 3), it can be used to predict changes of the state of the Baltic proper caused by reductions of the P supply. An inherent consequence of the formulation of the internal P source as proportional to the area of anoxic bottoms (Stigebrandt et al. 2014) is that oxygenation of anoxic bottoms shuts off the internal P source. This is also supported by extensive observational results from the Baltic proper and from the By Fjord presented in “Introduction” section. Shut off by oxygenation is also supported by the observation by Stigebrandt and Gustafsson (2007) that the P content of the Baltic proper decreased during the period 1985–1992 when deepwater bottoms were oxygenated by the surface layer when the top of the halocline was lowered from 60 to 100 m. Additional strong support is obtained by the rapid natural restorations of the Baltic proper that occurred in the past, provided they were due to sustained lowering of the halocline as suggested in the present paper. However, experience from lakes shows that restoration attempts by oxygenation often fail. In the thoroughly studied Lake Sempach and Lake Baldegg, the P concentrations at overturn were 150 and 520 mg m^−3^, respectively, which is 5 and 17 times higher than the Baltic proper winter water P concentration (ca 30 mg m^−3^). When artificially oxygenated, P retention on deeper lake sediments did not improve because the water/sediment interface remained anoxic due to unchanged high sedimentation rates (Gachter and Wehli 1998; Katsev and Dittrich 2013). This contrasts with the deep part of the Baltic proper where the sediment surface was quickly oxidized after an event of natural oxygenation by inflow of new deepwater (Rosenberg et al. 2016) and the P efflux during anoxic conditions were changed to P uptake (Hall et al. 2017). Much of the experience from highly eutrophicated lakes where restoration by oxygenation has failed is therefore not applicable to the mildly eutrophicated Baltic proper.

Defaunated anoxic and hypoxic bottoms may accumulate and store organic matter, e.g. Jessen et al. (2017). This accumulated organic matter is here called SOM—stored organic matter. During long-term artificial or natural oxygenation, the fraction δ of the earlier defaunated bottom area A_df_ may be covered by oxic water (O_2_ > 2 mL L^−1^) and re-colonized by benthic fauna while the remaining fraction (1-δ) will be covered by hypoxic water and remain defaunated. A crucial question is whether the activities of benthos in the re-colonized bottoms will create a new internal P source for the water column by releasing P enclosed in SOM (PSOM). Karlsson et al. (2010) described the re-colonization of benthic fauna of formerly dead bottoms in the inner Stockholm archipelago. Norkko et al. (2011) demonstrated that present-day *Marenzelleria*-bioturbated sediments in the Stockholm archipelago have a great capacity to store phosphorus which could lead to a net flux of P into the sediment. Ekeroth et al. (2016) collected bottom sediment from a long-term anoxic site at 150-m depth in the Baltic proper. The in situ benthic flux was 0.12 mmol P day^−1^ out from the sediment. Boxcosms were incubated in the laboratory for 74 days to follow the development of benthic fluxes to flow-through of oxygen-rich water. After 20 days, benthic macrofauna was added (*Marenzelleria* spp. and *Monoporeia affinis*). In the laboratory experiment, fluxes of DIP were weak and generally directed into the sediment and bottoms with Monoporeia affinis gave the largest P uptake by the sediment (Ekeroth et al. 2016). These essentially biogeochemical results suggest that earlier defaunated sediments may act as P sinks when recolonized. However, there is also a biological source/sink component. Stigebrandt et al., (2015a) discussed colonization and potential colonizers and suggested that demersal fish such as cod could benefit from the new food source. An unknown fraction α of the P contained in benthos in re-colonized bottoms will emanate from PSOM that thus will contribute the fraction α of the outflux of P to grazing demersal fish. The residual fraction (1 − *α*) comes from fresh organic matter. In a quasi-steady state, the fraction (1 − *β*) of the grazed P is brought on land by fisheries and the remainder, the fraction β, is excreted by the fish into the water column. The rate of excretion of PSOM by demersal fish is here considered to constitute the biologically executed component of the P source from recolonized bottoms. Among others, it depends on the area of the re-colonized bottoms *δ*·*A*_df_, the dominating benthic species, the fractions *α* and *β*, and the standing stocks of demersal fish. Estimation of the P source from re-colonized bottoms, defined by the sum of the rate of excretion of PSOM by demersal fish and the rate of P uptake by the re-colonized and oxidized sediment, as described above, is postponed to a forthcoming paper. A P source from re-colonized bottoms will contribute to Totsource and thereby, see Eq. (4), influence *c*_1e_ during a limited period until the SOM has been consumed.

Thanks to ambitious reductions of the external P supply since the 1980s, the present time land-based external P supply is about the same as that in the beginning of the 1950s, c.f. Stigebrandt et al. (2014). According to the P model in the present paper, restoration of the Baltic proper by keeping deep bottoms oxygenated would in 10–15 years change the trophic state from the present state to a state similar to that in the beginning of the 1950s, c.f. Fig. 6. Thereafter, the artificial oxygenation may stop and the Baltic proper should remain in the new state as long as the phosphorus supply does not increase by increased land-based external supply and/or by increased internal supply due to leakage from earlier defaunated bottoms and/or due to development of anoxic bottoms possibly initiated by major inflows of extremely salty new deepwater like in the 1950s. A possible P source from earlier defaunated bottoms, due to grazing demersal fish, may be reduced either by keeping *δ* small so that a large fraction (1 − *δ*) of the oxygenated bottoms are kept hypoxic (0 < O_2_ < 2 mL L^−1^) and defaunated or by a clever strategy to control grazing by controlling the magnitudes of the stocks of demersal fish. With all the experience that should be gathered during a restoration operation, it should in future be straightforward to prevent development of anoxia in the Baltic proper using oxygenation of the deepwater in critical basins when needed.

## Concluding remarks

The relationship between the equilibrium winter surface water P concentration *c*_1e_ and the total P supply, derived and verified in the present paper, makes it possible to predict changes of c_1e_ that should result due to changes of the total P supply. The equilibrium model should be a very important tool in the search for economically efficient measures to reduce the eutrophication of the Baltic proper which should be very attractive from a management point of view.

Sediment core data show that the deepwater sediments of the Baltic proper have alternated between oxygenated and anoxic episodes. To understand these switches, one must understand the water exchange of the Baltic proper and the resulting vertical stratification of the deepwater. Due to its topographical construction with a vast horizontal area outside the narrow and shallow entrance straits where out flowing Baltic proper surface water accumulates, the salinity of inflowing new deepwater varies extremely much, see Stigebrandt et al. (2015a) and references therein. Because of the huge variability, there may be long periods when deepwater inflows are relatively small and less saline permitting the upper layer of the Baltic proper to expand vertically which gives efficient oxygenation of the deep bottoms coming into contact with well-ventilated surface water. Oxygenation by a vertically expanded surface layer during the time TR is identified as the natural restoration mechanism, which is described and modeled in the present paper. This restoration mechanism has not earlier attracted much attention.

The P model in the present paper shows that by turning off the internal source, the Baltic proper may be restored in 10–15 years to a state in equilibrium with the land-based and oceanic external supplies. The predicted short restoration time is supported by the short termination time of anoxic periods as observed in sediment cores by Jilbert and Slomp (2013). The similarity between the Baltic Sea of today and of the anoxic period during the Medieval Climate Anomaly should be quite large and the model should be applicable to that period. However, it is not claimed here that the model is directly applicable to earlier anoxic periods when the similarity might be less due to topographic and volumetric differences caused by sea level changes and relative land rise, c.f. Jilbert et al. (2015).

It is also briefly discussed how restoration can be achieved by man-made oxygenation of the deepwater although the details are left for future investigations. When restoration is completed, the oxygenation equipment may be turned off. The likelihood that a restoration will last should be inversely proportional to the external P supply. It is therefore important that the land-based external P supply continues to be reduced.

The internal supply of phosphorus from anoxic bottoms is at present about three times greater than the land-based external supply (Stigebrandt et al. 2014). It is then easy to understand that even large cuts of the land-based external supply will have only minor effects as shown by the “BSAP-case” in the present paper, c.f. Fig. 6. If no action is taken to stop the internal supply, it is generally assumed that it may take a very long time before the phosphorus concentration will decrease. To predict this time is not possible since it depends on the appearance of prolonged periods of vertical expansion of the surface layer that give sustained oxygenation of deep bottoms, as suggested in the present paper.
